# No need to replace an “anomalous” primate (Primates) with an “anomalous” bear (Carnivora, Ursidae)

**DOI:** 10.3897/zookeys.487.9176

**Published:** 2015-03-16

**Authors:** Eliécer E. Gutiérrez, Ronald H. Pine

**Affiliations:** 1 Division of Mammals, National Museum of Natural History, NHB 390, MRC 108, Smithsonian Institution, P.O. Box 37012, Washington DC 20013-7012, USA; 2 Center for Conservation and Evolutionary Genetics, Smithsonian Conservation Biology Institute, National Zoological Park, Washington, DC 20008, USA; 3 Biodiversity Institute & Natural History Museum, University of Kansas, Lawrence, KS 66045 USA

**Keywords:** Mitochondrial DNA, phylogenetics, *Ursus maritimus*, *Ursus arctos*, Himalayas, yeti, cryptozoology

## Abstract

By means of mitochondrial 12S rRNA sequencing of putative “yeti”, “bigfoot”, and other “anomalous primate” hair samples, a recent study concluded that two samples, presented as from the Himalayas, do not belong to an “anomalous primate”, but to an unknown, anomalous type of ursid. That is, that they match 12S rRNA sequences of a fossil Polar Bear (*Ursus maritimus*), but neither of modern Polar Bears, nor of Brown Bears (*Ursus arctos*), the closest relative of Polar Bears, and one that occurs today in the Himalayas. We have undertaken direct comparison of sequences; replication of the original comparative study; inference of phylogenetic relationships of the two samples with respect to those from all extant species of Ursidae (except for the Giant Panda, *Ailuropoda melanoleuca*) and two extinct Pleistocene species; and application of a non-tree-based population aggregation approach for species diagnosis and identification. Our results demonstrate that the very short fragment of the 12S rRNA gene sequenced by Sykes et al. is not sufficiently informative to support the hypotheses provided by these authors with respect to the taxonomic identity of the individuals from which these sequences were obtained. We have concluded that there is no reason to believe that the two samples came from anything other than Brown Bears. These analyses afforded an opportunity to test the monophyly of morphologically defined species and to comment on both their phylogenetic relationships and future efforts necessary to advance our understanding of ursid systematics.

## Introduction

Sykes et al. (2014) conducted mitochondrial 12S rRNA sequencing on 30 hair samples from several geographic regions and that had been anecdotally attributed to “anomalous primates” (“yeti”, “almasty”, “orang pendek”, and “bigfoot”). All but two of these samples, both said to originate in the Himalayas, were identified by Sykes et al. as coming from domestic animals, well-known wild animals of the present day, or a human. Those two samples, however, were characterized as representing what could be termed an “anomalous carnivore” – a bear of the genus *Ursus*, with a “100% match with DNA recovered from a Pleistocene fossil more than [sic] 40 000 BP of *Ursus maritimus* (polar bear) […] but not to modern examples of the species”. In their text they noted that one of the hair samples was golden-brown and the other reddish-brown but also that white bears had been reported anecdotally from Central Asia and the Himalayas and that the genetic affinities of Himalayan bears are unknown. Sykes et al. stated that the hairs had been “thoroughly cleaned”, by some unspecified process, “to remove surface contamination”. We wonder if the hairs could have become discolored below the surface. One sample was said to be about 40 years old and the age of the other was unspecified, although Melton et al. (2015) stated that the latter had been represented as being about 10 years old. No information was given as to the conditions to which these hairs may have been exposed prior to the study by Sykes et al. These authors concluded that it seemed likely that the hairs were “from either a previously unrecognized bear species, colour variants of *Ursus maritimus*, or *Ursus arctos*/*Ursus maritimus* hybrids”, and, if hybrids, that they “are probably descended from a […] hybridization event during the early stages of species divergence between *Ursus arctos* and *Ursus maritimus*”. One of the samples was said to have come “from an animal shot by an experienced hunter […] who reported that its behavior was very different from [that of] a brown bear *Ursus arctos*, with which he was very familiar”. According to Sykes et al., “If these bears are widely distributed in the Himalayas, they may well contribute to the biological foundation of the yeti legend, especially if, as reported by the hunter who shot the […] specimen, they behave more aggressively toward humans than known indigenous bear species”. What strikes us as odd is that an “experienced hunter”, who was very familiar with the Brown Bear, could mistake the animal that he had shot for anything other than a bear of some sort and, specifically, for a “yeti”. Corroboration and documentation of, as well as other information concerning, the anecdote of this bear being shot by the hunter and the subsequent history of the hair that was saved would be most welcome. More documentation concerning the origin and subsequent history of the other sample, stated by the authors as having been “a nest of a migyhur, the Bhutanese equivalent of the yeti” would also be helpful. According to Edwards and Barnett (2015), rather than this sample being represented by a “nest”, it consisted only of a single hair. Although Sykes et al. take the accuracy of the stated locations for origination of the supposedly Himalayan bear hairs for granted, they reported certain tested samples from Russia as being from an American Black Bear (*Ursus americanus*) and a North American Raccoon (*Procyon lotor*), “even though they are native to North America”. The raccoon fur from Russia is not much of a surprise, because raccoons have been introduced, with varying degrees of success, to a variety of places in the former Soviet Union (Heptner et al. 2001, p. 1380).

According to various journalistic accounts (e.g. Hill 2014), “Sykes plans to […] organize an expedition to the Himalayas next year to look for a live specimen of the anomalous bear”. However, owing to uncertainties and omissions in the interpretations made by Sykes et al. of their data, we questioned their conclusion that there was reason to believe that there was some sort of bear, unknown to science, in the Himalayas. Accordingly, to test the inferences made by these authors, we carried out comparisons of 12S rRNA sequences of *Ursus maritimus* and *Ursus arctos* with the two bear sequences of Sykes et al.; replicated their comparison utilizing the Basic Local Alignment Search Tool (BLAST); conducted phylogenetic analyses incorporating sequences from the two specimens studied by Sykes et al. and of all extant species of Ursidae (except for the Giant Panda, *Ailuropoda melanoleuca*) and two extinct Pleistocene species; and employed the non-tree-based population aggregation approach for species diagnosis and identification. The phylogenetic analyses afforded an opportunity to test the monophyly of morphologically defined species and to comment on their phylogenetic relationships.

## Methods

Analyses we present herein were based on sequences of the mitochondrial 12S ribosomal RNA gene obtained from GenBank (www.ncbi.nlm.nih.gov/GenBank), some available as part of whole mitochondrial genomes from which we extracted them. GenBank accession numbers of these sequences are as follows: AB302321, AJ428577, AP012559–AP012597, AY012153, EF667005, EU327344, EU497665, FM177759, FM177760, FM177763–FM177765, FN390842–FN390859, FN390861–FN390872, GU573485–GU573491, JX196366–JX196392, KJ155697–KJ155699, KJ155710, KJ155713, KJ155717, KJ155718, KJ155722, KJ155723, KJ607607, L21882, L21884, L21889–L21891, NC011112, NC011116, NC011118, NC003426–NC003428, NC008753, NC009331, NC009968, NC009970, NC009971, U12854, U78349, Y08519, Y08520. We carried out direct comparisons of 12S rRNA sequences of the bear species *Ursus maritimus* and *Ursus arctos* with the two sequences produced and identified by Sykes et al. (2014) as representative of either “a previously unrecognized bear species, colour variants of *Ursus maritimus*, or *Ursus arctos*/*Ursus maritimus* hybrids.” These sequences are hereinafter referred to as “the focal sequences”. The GenBank accession numbers of these sequences are KJ155697 and KJ155722. These sequences were obtained from samples allegedly from India (ref. no. 25025 in Sykes et al. 2014) and Bhutan (ref. no. 25191), respectively. We also subjected the focal sequences to a BLAST analysis, thus replicating the comparison that Sykes et al. (2014) conducted against GenBank sequences. We conducted this analysis via the National Center of Biotechnology and Information’s website for standard BLAST of nucleotide sequences (http://www.ncbi.nlm.nih.gov/blast; Zhang et al. 2000, Morgulis et al. 2008), using the *blastn* method and the *nucleotide collection (nr/nt)* database, which we assume were also used by Sykes et al. (2014).

As an alternative method for taxonomic identification of the focal sequences, we inferred their phylogenetic relationships with respect to complete 12S rRNA sequences of representatives of the bear species *Helarctos malayanus* (3 sequences), *Melursus ursinus* (3), *Ursus americanus* (11), *Ursus arctos* (50), *Ursus maritimus* (32), *Ursus spelaeus*^†^ (33), and *Ursus thibetanus* (8). All of these species have been previously recovered in a well-supported monophyletic group sister to the bear species *Tremactos ornatus* (1) and *Arctodus simus*^†^ (1), both designated here as outgroups (Krause et al. 2008). Sequences were aligned using default options of MAFFT v.7.017 (Katoh et al. 2013) as implemented in Geneious v.7.1.5. We acknowledge that partitioning data for model-based phylogenetic analyses improves model fit by dividing alignments into relatively homogeneous sets of sites; however, for the purpose of this paper (primarily focused on the identity of the short focal sequences) we follow previous studies in which 12S rRNA data have not been subdivided (e.g. Lloyd 2003, Chambers et al. 2009, Westerman et al. 2012, Almeida et al. 2014). Thus, we used PartitionFinder ver. 1.0.1. (Lanfear et al. 2012) only for the purpose of determining the best-fit model of nucleotide substitution based on the corrected Bayesian Information Criterion (BIC). PartitionFinder considered only models that can be applicable in MrBayes.

Two optimality criteria were used for phylogenetic analyses, Bayesian inference (BI) and maximum likelihood (ML). The Bayesian topology was inferred with MrBayes v. 3. 2 (Ronquist et al. 2012). The search started with a random tree; the Markov chains ran for 100,000,000 generations, and trees were sampled every 1000 generations. The first 25,000 trees were discarded as burn-in, and the Bayesian posterior probability estimates were obtained based on the remaining 75,000 trees. The resulting parameter files were combined and assessed for stationarity and suitable effective sample size (ESS) values, using Tracer 1.6 (Rambaut et al. 2014). For this analysis, we consider as *strong* (*significant*) support only Bayesian posterior probability (BPP) values ≥ 0.95; as *moderate* (*nearly significant*) BPP values, those of 0.90–0.94; and as *negligible* BPP values, those of < 0.90. For obtaining the best topology under the ML criterion, we conducted 20 independent searches in the Genetic Algorithm for Rapid Likelihood Inference (GARLI 2.0; Zwickl 2006), using the default settings. Nodal support for the ML tree was assessed with nonparametric bootstrapping (Felsenstein 1985) in GARLI 2.0, based on 1000 searches (i.e. 100 pseudoreplicated data matrices and 10 searches for each of them). The degree of support received by individual nodes in the ML bootstrap analysis is referred to in the results and discussion sections by the following categories: strong support for bootstrap values ≥ 75%; moderate support for bootstrap values > 50% and < 75%; negligible support for values ≤ 50%.

Several studies have shown that a non-tree, character-based approach can help in species identifications that could not be accomplished with tree-based methods (DeSalle et al. 2005, Zou 2011, Van Velzen et al. 2012). Therefore, we also employed the non-tree-based population aggregation analysis approach (Davis and Nixon 1992) to detect if sequences of the 12S rRNA gene contain unique combinations of nucleotides that would allow diagnoses of *Ursus arctos* and *Ursus maritimus* and subsequent assignment of the focal sequences to either species.

## Results

### Sequence comparisons and BLAST

Comparisons of the two sequence fragments of the mitochondrial 12S ribosomal RNA gene produced by Sykes et al. (2014) with homologous fragments for *Ursus arctos* (49 individuals) and *Ursus maritimus* (32 individuals) revealed few and inconsistent differences between these species. The focal sequences are identical to each other and possess a length of 104 base pairs, which correspond to positions 451–554 in complete sequences of the 12S rRNA gene (using as reference GenBank sequence NC003428). Four positions show differences between *Ursus arctos* and *Ursus maritimus*, with the former species being polymorphic in three positions, and the latter only in one (Table 1). The focal sequences differ with respect to most other sequences in two positions (Table 1). In position 474 (position 24 of fragment), one *Ursus maritimus* and all but nine *Ursus arctos* match the focal sequences in having a thymine. In position 550 (100 of fragment), all *Ursus maritimus* and nine *Ursus arctos* match the focal sequences in having a thymine. In the other two variable sites (positions 478 and 492), the nucleotide of the majority of sequences of both species matches that of the focal specimens (Table 1). The BLAST run retrieved as best matches of the focal sequences, two sequences of *Ursus maritimus* with hit scores of 193. With slightly lower hit scores (187), the second-best matches were 98 sequences, of which 94 corresponded to *Ursus arctos* and four to *Ursus maritimus*.

**Table 1.** Nucleotide variability of the fragment sequences of the 12S rRNA gene herein analyzed. Differences found in comparisons of the two fragment sequences (104 base-pairs long) produced by Sykes et al. (2014), and referred to in the text as focal sequences, with homologous fragments for *Ursus arctos* (49 individuals) and *Ursus maritimus* (32 individuals). The two focal sequences are identical to each other. The compared fragments correspond to positions 451–554 in complete sequences of gene 12S rRNA (using as reference GenBank sequence NC003428). Number of individuals is shown within parentheses.

Species	Corresponding positions in complete 12S gene sequences			
	474	478	492	550
*Ursus arctos*	T (40); C (9)	A (44); G (5)	A (1); G (48)	T (9); C (40)
*Ursus maritimus*	T (1); C (31)	A (32)	G (32)	T (32)
Focal sequences	T	A	G	T

### Tree and non-tree, character-based approach

**Figure 1.**
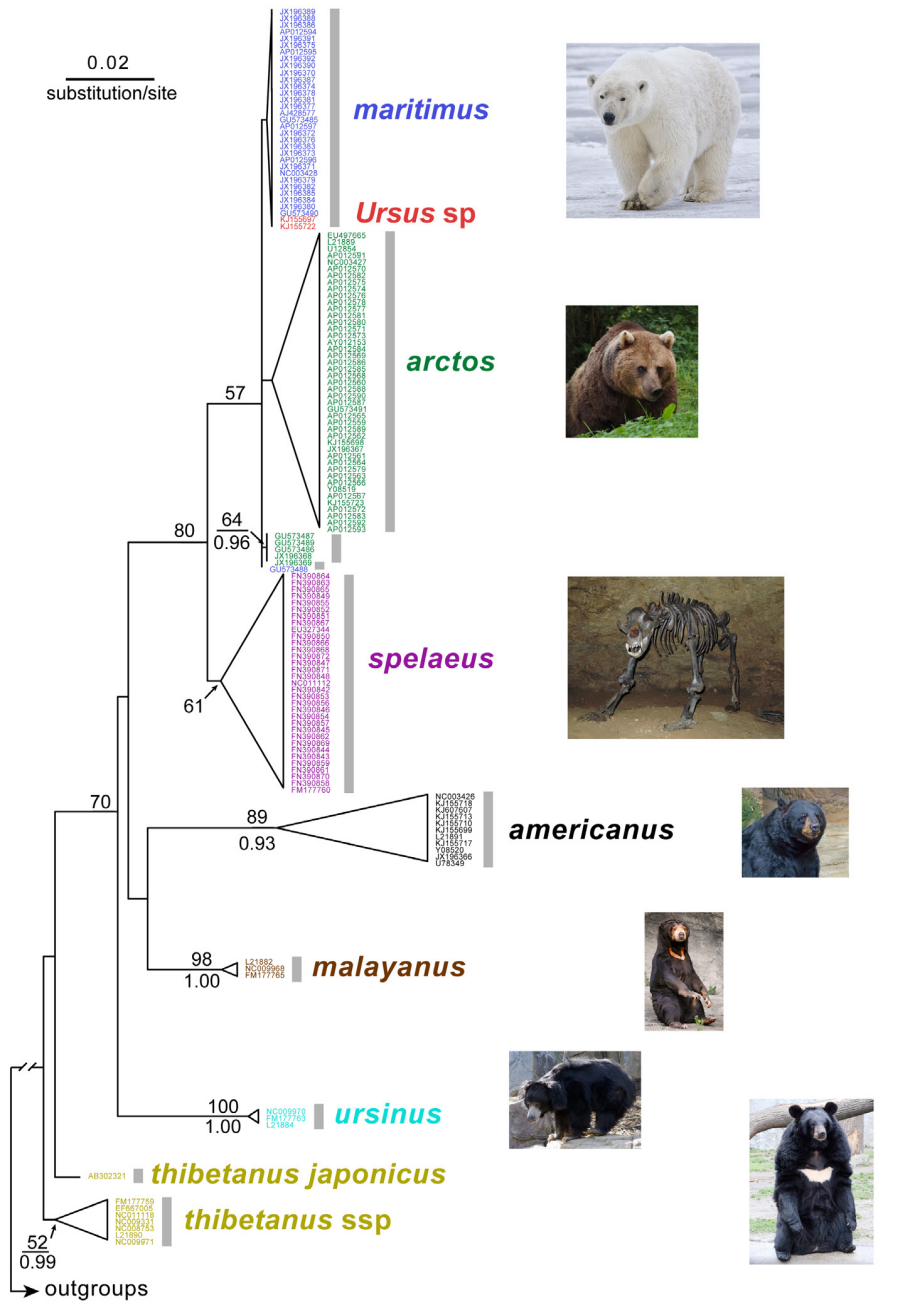
The maximum-likelihood tree resulting from the analysis of sequence data for the mitochondrial 12S ribosomal RNA gene under its best–fitting model (GTR + G, ln-likelihood - 3123.29336). Only non-negligible nodal support is indicated. Bootstrap values for the maximum-likelihood analysis are indicated above branches, whereas Bayesian posterior probabilities are indicated below branches. See Acknowledgments for photo credits.

The maximum likelihood (ML) analysis recovered the focal sequences in a large haplogroup containing sequences of *Ursus maritimus* and *Ursus arctos* (Figure 1), which received moderate bootstrap support. Three haplogroups were recovered within it: (a) one containing the focal sequences and all but one sequence of *Ursus maritimus*, (b) one containing most sequences of *Ursus arctos*, and (c) one containing five sequences of *Ursus arctos*. Only the latter haplogroup received non-negligible support. These three haplogroups formed a polytomy in which an additional sequence of *Ursus maritimus* was also found (not nested within either of the three haplogroups just described). The extinct *Ursus spelaeus* was recovered sister to this large haplogroup (*Ursus maritimus* + *Ursus arctos* + focal sequences) with strong support; however, the monophyly of *Ursus spelaeus* received only moderate bootstrap support. The clade containing the focal sequences, *Ursus maritimus*, *Ursus arctos*, and *Ursus spelaeus* was recovered sister, although with negligible support, to a clade containing a sister species pair formed by *Ursus americanus* and *Helarctos malayanus*. The latter two species were recovered as monophyletic, each with strong support. All of the species mentioned so far formed an unsupported clade that was recovered sister to *Melursus ursinus*, with moderate support. The monophyly of the latter species was moderately supported. All these taxa formed a clade that received negligible support, and which appeared sister to one sequence of *Ursus thibetanus japonicus*. All other sequences of *Ursus thibetanus* formed a moderately supported haplogroup sister to the rest of the ingroup. The ingroup was recovered monophyletic albeit with negligible support.

The Bayesian inference (BI) analysis yielded an even less resolved tree (topology not shown; but see nodal supports overimposed on ML tree in Figure 1). The major difference with respect to the ML tree was the position of *Ursus americanus*, found monophyletic, with strong support, sister to a clade containing all other ingroup taxa. The latter clade received negligible support, and its internal topology lacked resolution. By contrast with the ML tree, in the BI tree the focal sequences did not appear most closely related to any particular haplogroup or individual sequence. In this very large polytomy, only *Melursus ursinus*, *Helarctos malayanus*, and *Ursus thibetanus* were recovered monophyletic with strong nodal support.

The application of the non-tree-based population aggregation analysis (Davis and Nixon 1992) did not yield results that would allow identification of the focal sequences. In inspection of complete sequences of the 12S rRNA gene, we found no combination of nucleotides (and not even a single nucleotide) that would allow diagnosing *Ursus arctos* and *Ursus maritimus*.

## Discussion

### No evidence of a taxonomically unrecognized bear in the Himalayas

The molecular data obtained and analyzed by Sykes et al. (2014) are not informative enough to suggest the possibility that a taxonomically unrecognized type of bear exists in the Himalayas. The interpretations made by Sykes et al. with respect to the possible taxonomic identity of the focal sequences were based merely on the results of a BLAST run against GenBank sequences. The short fragment sequences of the mitochondrial 12S rRNA gene obtained by Sykes et al. from their hair samples and homologous fragment sequences of *Ursus arctos* and *Ursus maritimus* are all identical except in four positions. In these four positions either one or both known species are polymorphic, and in all of them at least some individuals of both species have the same nucleotide. More importantly, because in complete sequences of the 12S rRNA gene not even a single nucleotide consistently allows discrimination between *Ursus arctos* and *Ursus maritimus*, it is impossible to unambiguously assign a taxonomic identification, based on this gene, to the focal sequences. The results of a BLAST analysis could not rule out the possibility that the focal sequences belong to *Ursus arctos*, which is known to occur in the Himalayas. In congruence with this fact, the results of our BLAST analysis showed that numerous sequences of both *Ursus maritimus* and *Ursus arctos* yielded highly similar hit scores to those with the highest hit score, two sequences of *Ursus maritimus* (see Results). Unfortunately, Sykes et al. did not report any hit scores resulting from their analyses.

Our phylogenetic analyses do not provide evidence to rule out the possibility that the focal sequences might belong to *Ursus arctos*. Although in the ML analysis the focal sequences were recovered within a haplogroup with part of the sequences of *Ursus maritimus*, this haplogroup received negligible support. If we consider only relationships that received either moderate or strong nodal support, then not even the haplogroups containing sequences of *Ursus arctos* and *Ursus maritimus* would be distinguished from each other. This is an expected result considering that male-mediated dispersal and sex-biased gene flow have been reported between *Ursus arctos* and *Ursus maritimus* (Nakagome et al. 2008, Cahill et al. 2013 and 2014, Cronin et al. 2013, Bidon et al. 2014, Liu et al. 2014). Our phylogenetic analyses indicate that even complete sequences of the 12S rRNA gene do not consistently recover with moderate or strong support the monophyly of other ursid species, despite their monophyly having been established in previous studies. Based on mitochondrial DNA, previous studies found *Ursus arctos* paraphyletic with respect to *Ursus maritimus* and suggested past hybridization or incomplete lineage sorting as possible explanations (Bon et al. 2008, Lindqvist et al. 2010, Miller et al. 2012, Cronin et al. 2013, Hirata et al. 2013 and 2014; see also Hailer et al. 2013). Nevertheless, analyses of amplified fragment length polymorphisms (AFLP) data recovered *Ursus arctos* and *Ursus maritimus* as reciprocally monophyletic species (Cronin et al. 2013), and additional analyses of ca. 9100 nucleotides from 14 independent nuclear loci across the genome of 45 individuals yielded the same result, while also dating the split from the common ancestor of this sister-species pair as far back as from the Middle Pleistocene (Hailer et al. 2012; see also Kutschera et al. 2014, Liu et al. 2014). Given that hybridization between *Ursus arctos* and *Ursus maritimus* has been documented (see citations above), and that this precludes analyses of mitochondrial DNA being capable of recovering their reciprocal monophyly (even when complete mitochondrial genomes are analyzed; Bon et al. 2008, Lindqvist et al. 2010), it could not be expected that the fragment of the 12S rRNA gene sequenced by Sykes et al. would contain phylogenetic signal that could rule out the possibility that the focal sequences belong to *Ursus arctos*. Sykes et al. did not consider this possibility, and lacked evidence to support the scenarios they considered likely – i.e. that the focal sequences belong to individuals of “a previously unrecognized bear species, colour variants of *Ursus maritimus*, or *Ursus arctos*/*Ursus maritimus* hybrids”. The most parsimonious hypothesis regarding the identity of the Himalayan samples of Sykes et al. is that they are from *Ursus arctos*.

Based on different methods, Edwards and Barnett (2015) have recently concluded that the focal sequences most likely belong to *Ursus arctos isabellinus*. However, in the absence of modern revisionary work delimiting subspecies of *Ursus arctos*, we consider it appropriate to refer to Himalayan populations of the Brown Bear simply as *Ursus arctos*, and to take the opportunity to call the attention of the community of mammalian systematists to the need for such studies. In their study, Edwards and Barnett (2015) also demonstrated that the focal 104-bp long sequences do not match a homologous fragment of ancient *Ursus maritimus*, as asserted by Sykes et al. (2014), but rather that of a recent sample of that species, from Diomede, Little Diomede Island, Alaska (GenBank accession number GU573490). In addition, these authors suggested that the sequences produced by Sykes et al. (2014) could have resulted from artifacts due to the use of degraded DNA obtained from samples that were not freshly preserved, but Melton et al. (2015) consider this possibility unlikely.

Because financial and human resources are limited, it is necessary that they are invested in addressing well-founded scientific questions. If further resources were to be invested in determining the taxonomic identity of the bear populations from the Himalayas, the first step should be to obtain nuclear sequence data from museum specimens from that region. A query through the Global Biodiversity Information Facility (GBIF; http://www.gbif.org) indicated that at least 16 museum specimens identified as being of *Ursus arctos* from the Himalayas and nearby areas are housed in four North American and one European institution, namely the American Museum of Natural History, the Field Museum, the Museum of Comparative Zoology of Harvard University, the National Museum of Natural History, and the Natural History Museum of Geneva. Because many institutions do not yet share their data through GBIF, we expect that more specimens are readily available. Pyrosequencing has made the use of museum specimens to obtain large amounts of DNA sequence data a common, reliable practice (see Guschanski et al. 2013, Fabre et al. 2014). In our opinion, the use of pyrosequencing would be preferable to meeting the high costs of an expedition to attempt to obtain a freshly preserved tissue sample from a living animal (with no guarantee of success). By contrast, Melton et al. (2015; see also Hill 2014) seem to advocate for conducting an expedition. Aside from this methodological consideration, we emphasize that no evidence has ever been presented to suggest that an unknown bear species occurs in the Himalayas (contra Sykes et al. 2014 and Melton et al. 2015).

### Species monophyly and interspecific phylogenetic relationships

Although it is necessary to employ data obtained from multiple, independently inherited sources (e.g. sequence data from mtDNA and nDNA from different chromosomes) in order to reliably infer interspecific phylogenetic relationships, the gene tree resulting from our analyses provides insights on species monophyly and interspecific relationships that might be useful in planning future studies on bear systematics. In this regard, it is noteworthy that by using the 12S rRNA gene, we were able to analyze more individuals per species than has been done in previous studies, thus allowing us to assess both whether a deeper sampling enables detection of relationships previously unreported and to test whether morphologically defined species are recovered as monophyletic. We limit our discussion to relationships that received non-negligible support from either of the two analyses conducted.

Four out of the seven species of our ingroup were recovered in monophyletic haplogroups, the exceptions being *Ursus arctos*, *Ursus maritimus*, and *Ursus thibetanus*. The fact that neither of the first two species was recovered as monophyletic might result from possible ancestral polymorphism or past hybridization events (see Edwards et al. 2011, Cahill et al. 2013 and 2014; and citations above). On the other hand, the non-monophyly of *Ursus thibetanus* resulted from the exclusion of a single sequence, representing an individual of *Ursus thibetanus japonicus*, from a strongly supported haplogroup that included all other sequences of *Ursus thibetanus*. This result might be of taxonomic interest because populations of *Ursus thibetanus japonicus* might have experienced substantial isolation from mainland populations of *Ursus thibetanus*.

With regard to interspecific relationships, in congruence with results from numerous previous studies (e.g. Bon et al. 2008, Lindqvist et al. 2010, Cronin et al. 2013), our analyses found *Ursus arctos* and *Ursus maritimus* to form a monophyletic group; however, the internal topology of this clade was not resolved, which is not surprising, given instances of gene flow between these two species (see citations above) and that their reciprocal monophyly was recovered only with the use of nuclear data (Hailer et al. 2012, Cronin et al. 2013, Kutschera et al. 2014, Liu et al. 2014; see also Hailer et al. 2013). Similarly, the sister relationship between the clade just described (i.e. *Ursus arctos* + *Ursus maritimus*) and the extinct *Ursus spelaeus* was also found in other studies in which *Ursus spelaeus* was included (Bon et al. 2008, Krause et al. 2008). In addition, we recovered *Melursus ursinus* as sister to an (unsupported) clade that included all other ingroup species except *Ursus thibetanus*. This position for *Melursus ursinus* is incongruent with results from previous studies, which placed the species as sister to either all of our ingroup species (Yu et al. 2004 p. 488, Bon et al. 2008, Krause et al. 2008) or to *Ursus malayanus* (Li et al. 2004 (p. 486), Kutschera et al. 2014). Similarly, *Ursus thibetanus* (excluding *Ursus thibetanus japonicus*) was found to be sister to the rest of the ingroup of our gene tree. This position is incongruent with results of previous studies that have found the species either sister to *Ursus americanus*, based on mtDNA (Krause et al. 2008), or *Ursus malayanus*, based on nDNA (Kutschera et al. 2014).

Despite conflicting results among studies that have looked at interspecific phylogenetic relationships, we believe that further efforts on ursid systematics should also focus on assessing geographic variation, phylogeographic patterns of widespread species, and taxonomy. The hypothetical non-monophyletic nature of *Ursus thibetanus*, exposed in our results, represents an example of the kinds of problems meriting attention by taxonomists. The use of DNA from museum specimens and techniques such as Restriction site Associated DNA (RAD) tags that gather data from throughout genomes could significantly advance our understanding of this recent carnivore radiation, including clarification of the taxonomic position of nominal forms that have never been subjected to a modern systematic revision.

## References

[B1] Almeida FC Giannini NP Simmons NB Helgen KM (2014) Each flying fox on its own branch: a phylogenetic tree for *Pteropus* and related genera (Chiroptera: Pteropodidae). Molecular Phylogenetics and Evolution 77: 83–95. doi: 10.1016/j.ympev.2014.03.009 24662680 10.1016/j.ympev.2014.03.009

[B2] Bidon T Janke A Fain SR Eiken HG Hagen SB Saarma U Hallström BM Lecomte N Hailer F (2014) Brown and polar bear Y chromosomes reveal extensive male-biased gene flow within brother lineages. Molecular Biology and Evolution 31(6): 1353–1363. doi: 10.1093/molbev/msu109 24667925 10.1093/molbev/msu109

[B3] Bon C Caudy N de Dieuleveult M Fosse P Philippe M Maksud F Beraud-Colomb E Bouzaide E Kefie R Laugier C Rousseau B Casane D van der Plicht J Elalouf J-M (2008) Deciphering the complete mitochondrial genome and phylogeny of the extinct cave bear in the Paleolithic painted cave of Chauvet. Proceedings of the National Academy of Science of the United States of America 105(45): 17447–17452. doi: 10.1073/pnas.0806143105, www.pnas.org/cgi/doi/10.1073/pnas.0806143105 10.1073/pnas.0806143105 PMC2582265 18955696

[B4] Cahill JA Green RE Fulton TL Stiller M Jay F et al. (2013) Genomic evidence for island population conversion resolves conflicting theories of polar bear evolution. PLoS Genetics 9(3): . doi: 10.1371/journal.pgen.1003345 10.1371/journal.pgen.1003345 PMC3597504 23516372

[B5] Cahill JA Stirling I Kistler L Salamzade R Ersmark E Fulton TL Stiller M Green RE Shapiro B (2014) Genomic evidence of geographically widespread effect of gene flow from polar bears into brown bears. Molecular Ecology. doi: 10.1111/mec.13038 10.1111/mec.13038 PMC4409089 25490862

[B6] Cronin MA McDonough MM Huynh HM Baker RJ (2013) Genetic relationships of North American bears (*Ursus*) inferred from amplified fragment length polymorphisms and mitochondrial DNA sequences. Canadian Journal of Zoology 91: 626–634. doi: 10.1139/cjz-2013-0078

[B7] Davis JI Nixon KC (1992) Populations, genetic variation, and the delimitation of phylogenetic species. Systematic Biology 41: 421–435. doi: 10.1093/sysbio/41.4.421

[B8] DeSalle R Egan MG Siddall M (2005) The unholy trinity: taxonomy, species delimitation and DNA barcoding. Philosophical Transactions of the Royal Society B: Biological Sciences 360(1462): 1905–1916. doi: 10.1098/rstb.2005.1722 10.1098/rstb.2005.1722 PMC1609226 16214748

[B9] Edwards CJ Barnett R (2015) Himalayan ‘yeti’ DNA: polar bear or DNA degradation? A comment on ‘Genetic analysis of hair samples attributed to yeti’ by Sykes et al. (2014). Proceedings of the Royal Society of London B 282: . doi: 10.1098/rspb.2014.1712 10.1098/rspb.2014.1712 PMC4298200 25520353

[B10] Edwards CJ Suchard MA Lemey P Welch JJ Barnes I Fulton TL Barnett R O’Connell TC Coxon P Monaghan N Valdiosera CE Lorenzen ED Willerslev E Baryshnikov GF Rambaut A Thomas MG Bradley DG Shapiro B (2011) Ancient hybridization and an Irish origin for the modern polar bear matriline. Current Biology 21: 1251–1258. doi: 10.1016/j.cub.2011.05.058 21737280 10.1016/j.cub.2011.05.058 PMC4677796

[B11] Fabre P-H Vilstrup JT Raghavan M Der Sarkissian C Willerslev E Douzery EJP Orlando L (2014) Rodents of the Caribbean: origin and diversification of hutias unravelled by next-generation museomics. Biology Letters 10: . doi: 10.1098/rsbl.2014.0266 10.1098/rsbl.2014.0266 PMC4126619 25115033

[B12] Felsenstein J (1985) Confidence limits on phylogenies: an approach using the bootstrap. Evolution 39: 783–791. doi: 10.2307/2408678 10.1111/j.1558-5646.1985.tb00420.x 28561359

[B13] Guschanski K Krause J Sawyer S Valente LM Bailey S Finstermeier K Sabin R Gilissen E Sonet G Nagy ZT Lenglet G Mayer F Savolainen V (2013) Next-generation museomics disentangles one of the largest primate radiations. Systematic Biology 62(4): 539–554. doi: 10.1093/sysbio/syt018 23503595 10.1093/sysbio/syt018 PMC3676678

[B14] Hailer F Kutschera VE Hallström BM Klassert D Fain SR Leonard JA Arnason U Janke A (2012) Nuclear genomic sequences reveal that polar bears are an old and distinct bear lineage. Science 336(6079): 344–347. doi: 10.1126/science.1216424 22517859 10.1126/science.1216424

[B15] Hailer F Kutschera VE Hallström BM Klassert D Fain SR Leonard JA Arnason U Janke A (2013) Response to comment on “Nuclear genomic sequences reveal that polar bears are an old and distinct bear lineage”. Science 339(6127): . doi: 10.1126/science.1228066 10.1126/science.1228066 23539581

[B16] Heptner VG Naumov NP Yurgenson PB Sludskii AA Chirkova AF Bannikov AG (2001) Mammals of the Soviet Union. Volume II, Part lb. Carnivora (Weasels; Additional Species). Smithsonian Institution Libraries and The National Science Foundation, Washington DC, 1552 pp.

[B17] Hill S (2014) Bigfoot DNA disappoints and reveals surprise. Skeptical Inquirer 38(6): 6–7.

[B18] Hirata D Mano T Abramov A Baryshnikov G Kosintsev P Vorobiev A Raichev E Tsunoda H Kaneko Y Murata K Fukui D Masuda R (2013) Molecular phylogeography of the brown bear (*Ursus arctos*) in northeastern Asia based on analyses of complete mitochondrial DNA sequences. Molecular Biology and Evolution 30: 1644–1652. doi: 10.1093/molbev/mst077 23619144 10.1093/molbev/mst077

[B19] Hirata D Abramov AV Baryshnikov GF Masuda R (2014) Mitochondrial DNA haplogrouping of the brown bear, *Ursus arctos* (Carnivora: Ursidae) in Asia, based on a newly developed APLP analysis. Biological Journal of the Linnean Society 111: 627–635. doi: 10.1111/bij.12219

[B20] Katoh K Standley DM (2013) MAFFT Multiple Sequence Alignment Software version 7: improvements in performance and usability. Molecular Biology and Evolution 30: 772–780. doi: 10.1093/molbev/mst010 23329690 10.1093/molbev/mst010 PMC3603318

[B21] Krause J Unger T Noçon A Malaspinas A-S Kolokotronis S-O Stiller M Soibelzon L Spriggs H Dear PH Briggs AW Bray SCE O’Brien SJ Rabeder G Matheus P Cooper A Slatkin M Pääbo S Hofreiter M (2008) Mitochondrial genomes reveal an explosive radiation of extinct and extant bears near the Miocene–Pliocene boundary. BMC Evolutionary Biology 8: . doi: 10.1186/1471-2148-8-220 10.1186/1471-2148-8-220 PMC2518930 18662376

[B22] Kutschera VE Bidon T Hailer F Rodi JL Fain SR Janke A (2014) Bears in a forest of gene trees: phylogenetic inference is complicated by incomplete lineage sorting and gene flow. Molecular Biology and Evolution 31(8): 2004–2017. doi: 10.1093/molbev/msu186 24903145 10.1093/molbev/msu186 PMC4104321

[B23] Lanfear R Calcott B Ho SYW Guindon S (2012) PartitionFinder: combined selection of partitioning schemes and substitution models for phylogenetic analyses. Molecular Biology and Evolution 29: 1695–1701. doi: 10.1093/molbev/mss020 22319168 10.1093/molbev/mss020

[B24] Lindqvist C Schuster SC Sun Y Talbot SL Qi J Ratan A Tomsho LP Kasson L Zeyl E Aars J Miller W Ingólfsson Ó Bachmann L Wiig Ø (2010) Complete mitochondrial genome of a Pleistocene jawbone unveils the origin of polar bear. Proceedings of the National Academy of Science of the United States of America 107: 5053–5057. doi: 10.1073/pnas.0914266107 10.1073/pnas.0914266107 PMC2841953 20194737

[B25] Liu S Lorenzen ED Fumagalli M Li B Harris K Xiong Z Zhou L Korneliussen TS Somel M Babbitt C Wray G Li J He W Wang Z Fu W Xiang X Morgan CC Doherty A O’Connell MJ McInerney JO Born EW Dalén L Dietz R Orlando L Sonne C Zhang G Nielsen R Willerslev E Wang J (2014) Population genomics reveal recent speciation and rapid evolutionary adaptation in polar bears. Cell 157(4): 785–794 doi: 10.1016/j.cell.2014.03.054 24813606 10.1016/j.cell.2014.03.054 PMC4089990

[B26] Lloyd BD (2003) Intraspecific phylogeny of the New Zealand short-tailed bat *Mystacina tuberculata* inferred from multiple mitochondrial gene sequences. Systematic Biology 52(4): 460–476. doi: 10.1080/10635150390218187 12857638 10.1080/10635150390218187

[B27] Melton TW Sartori M Sykes BC (2015) Response to Edward [sic] and Barnett. Proceedings of the Royal Society of London B 282: . doi: 10.1098/rspb.2014.2434 10.1098/rspb.2014.2434 PMC4298211 25520360

[B28] Miller W Schuster SC Welch AJ Ratan A Bedoya-Reina OC Zhao F Kim HL Burhans RC Drautz DI Wittekindt NE Tomsho LP Ibarra-Laclette E Herrera-Estrella L Peacock E Farley S Sage GK Rode K Obbard M Montiel R Bachmann L Ingólfsson Ó Aars J Mailund T Wiig Ø Talbot SL Lindqvist C (2012) Polar and brown bear genomes reveal ancient admixture and demographic footprints of past climate change. Proceedings of the National Academy of Science of the United States of America 109(36): E2382–E2390. doi: 10.1073/pnas.1210506109 10.1073/pnas.1210506109 PMC3437856 22826254

[B29] Morgulis A Coulouris G Raytselis Y Madden TL Agarwala R Schäffer AA (2008) Database indexing for production MegaBLAST searches. Bioinformatics 24: 1757–1764. doi: 10.1093/bioinformatics/btn322 18567917 10.1093/bioinformatics/btn322 PMC2696921

[B30] Nakagome S Pecon-Slattery J Masuda R (2008) Unequal rates of Y chromosome gene divergence during speciation of the family Ursidae. Molecular Biology and Evolution 25(7): 1344–1356. doi: 10.1093/molbev/msn086 18400788 10.1093/molbev/msn086

[B31] Rambaut A Suchard MA Xie D Drummond AJ (2014) Tracer v1.6. http://beast.bio.ed.ac.uk/Tracer

[B32] Ronquist F Teslenko M van der Mark P Ayres DL Darling A Höhna S Larget B Liu L Suchard MA Huelsenbeck JP (2012) MrBayes 3.2: efficient Bayesian phylogenetic inference and model choice across a large model space. Systematic Biology 61: 539–542. doi: 10.1093/sysbio/sys029 22357727 10.1093/sysbio/sys029 PMC3329765

[B33] Sykes BC Mullis RA Hagenmuller C Melton TW Sartori M (2014) Genetic analysis of hair samples attributed to yeti, bigfoot and other anomalous primates. Proceedings of the Royal Society B 281: . doi: 10.1098/rspb.2014.0161 10.1098/rspb.2014.0161 PMC4100498 24990672

[B34] Van Velzen R Weitschek E Felici G Bakker FT (2012) DNA barcoding of recently diverged species: relative performance of matching methods. PLoS ONE 7(1): . doi: 10.1371/journal.pone.0030490 10.1371/journal.pone.0030490 PMC3260286 22272356

[B35] Westerman M Kear BP Aplin K Meredith RW Emerling C Springer MS (2012) Phylogenetic relationships of living and recently extinct bandicoots based on nuclear and mitochondrial DNA sequences. Molecular Phylogenetics and Evolution 62: 97–108. doi: 10.1016/j.ympev.2011.09.009 22100729 10.1016/j.ympev.2011.09.009

[B36] Yu L Li Q-W Ryder OA Zhang Y-P (2004) Phylogeny of the bears (Ursidae) based on nuclear and mitochondrial genes. Molecular Phylogenetics and Evolution 32: 480–494. doi: 10.1016/j.ympev.2004.02.015 15223031 10.1016/j.ympev.2004.02.015

[B37] Zhang Z Schwartz S Wagner L Miller W (2000) A greedy algorithm for aligning DNA sequences. Journal of Computational Biology 7(1–2): 203–214. doi: 10.1089/10665270050081478 10890397 10.1089/10665270050081478

[B38] Zou S Li Q Kong L Yu H Zheng X (2011) Comparing the usefulness of distance, monophyly and character-based DNA barcoding methods in species identification: a case study of Neogastropoda. PLoS ONE 6(10): . doi: 10.1371/journal.pone.0026619 10.1371/journal.pone.0026619 PMC3200347 22039517

[B39] Zwickl DJ (2006) Genetic algorithm approaches for the phylogenetic analysis of large biological sequence datasets under the maximum likelihood criterion. PhD thesis, University of Texas, Austin.

